# From fire to ashes. A six-month follow-up prospective study of manic patients after acute inward hospitalisation

**DOI:** 10.1192/j.eurpsy.2025.1084

**Published:** 2025-08-26

**Authors:** S. Salmeron, J. I. Mena, E. Cesari, H. Andreu, L. Olivier, O. de Juan, B. Serra, I. Ochandiano, A. Giménez-Palomo, I. Pacchiarotti

**Affiliations:** 1 Psychiatry and Psychology Service, Hopsital Clínic de Barcelona; 2 Institut de Recerca Biomèdica August Pi i Sunyer (IDIBAPS), Barcelona, Spain

## Abstract

**Introduction:**

Although manic episodes are the most characteristic and diagnostic, depressive episodes and subsyndromal depressive symptoms are most prevalent through bipolar patients lifetime and are most related to functional impairment. Manic episodes are still more frequently psychiatrically identified and managed and represent for many patients the first contact with mental health professional and the beginning of an adequate follow-up. They are, however, frequently followed by depressive states. For these reasons, post-episodic constitutes an important psychiatric task for monitoring and follow-up, with little evidence supporting its risks and needs.

**Objectives:**

To elucidate the sociodemographic and clinical characteristics related to depressive switch of bipolar disorder patients after hospital discharge for a manic episode.

**Methods:**

This is a prospective study involving consecutive patients admitted for a manic episode in acute inward in Hospital Clínic de Barcelona. Patients were recruited upon admission to the hospital and followed for a period of six months. Clinical evaluations were conducted during ospital admission and at discharge with Brief Psychiatric Rating Scale, Young Mania Rating Scale, Hamilton Depression Rating Scale and Clinical Global Impression scale for Bipolar Disorder. Patients were contacted for follow-up assessments at 1 month and 6 months after discharge. Presence of a depressive episode was evaluated using the DSM-5 criteria.

**Results:**

A total of 46 patients were recruited, of whom 17 (37%) presented a depressive episode in the following 6 months after discharge. In relation to psychiatric history, manic predominance showed a significant negative association with depressive switch (p=0.004), with depressive predominance showing and approached significance for a positive association (p=0.097). Also, schizoaffective disorder diagnosis approached significance for a negative association (p=0.071). Comorbidity with substance use disorder (except tobacco) showed significant positive association (p=0.417). Lithium is the only treatment showing a significant positive association with depressive switch (p=0.046).

**Conclusions:**

Lack of significance for clinical variables suggest no clear capacity for predicting follow-up evolution through psychopathological state at discharge. Manic and depressive predominance show, respectively, a negative and positive association with depressive switch, maintaining the global tendency of individual patient’s disorder. Interestingly, comorbidity with substance use disorder acted as a protective factor for depressive switching. This finding might be in relation with the fact that, whereas in substance users drugs act as a main factor for their psychopathological state and evolution, with a proneness for stimulants towards manic states, non-users present a higher degree of endogeneity, where depressive states constitute the main tendency.

**Disclosure of Interest:**

S. Salmeron: None Declared, J. I. Mena: None Declared, E. Cesari: None Declared, H. Andreu: None Declared, L. Olivier: None Declared, O. de Juan: None Declared, B. Serra: None Declared, I. Ochandiano: None Declared, A. Giménez-Palomo Consultant of: AGP has received CME-related honoraria, or consulting fees from Angelini, Janssen-Cilag, Casen Recordati, Rovi, LCN and Lundbec, I. Pacchiarotti Consultant of: IP has received CME-related honoraria, or consulting fees from Janssen-Cilag, Lundbeck, undbeck/Otsuka, CASEN Recordati and Angelini, with no financial or other relationship relevant to the subject of this article.

